# Spontaneous Calcium Signalling in the Developing Mammalian Cochlea

**DOI:** 10.1007/s10162-026-01035-1

**Published:** 2026-02-23

**Authors:** Federico Ceriani, Walter Marcotti

**Affiliations:** 1 School of Biosciences, University of Sheffield, S10 2TN, Sheffield, UK; 2 Neuroscience Institute, University of Sheffield, S10 2TN, Sheffield, UK

**Keywords:** Cochlea, Hair cells, Supporting cells, Auditory pathway, Spiral ganglion neurons, Purinergic signalling, Development, In vivo imaging, Spontaneous activity

## Abstract

In mammals, the accurate and high-fidelity representation of sound largely depends on the cochlea, the sensory organ specialized for transducing acoustic signals into neural activity with remarkable temporal precision. Prior to hearing onset, which occurs around postnatal day 12 in most altricial rodents, the immature cochlea plays an active role in the refinement of neural circuits along the auditory pathway. To accomplish this function, sensory hair cells and glia-like supporting cells in the immature cochlea generate distinct patterns of spontaneous Ca^2+^ signals. Synchronized Ca^2+^-dependent activity across multiple hair cells is conveyed to the ascending auditory neurons, where it contributes to the emergence of tonotopic maps that enable frequency discrimination. Within the cochlea itself, this spontaneous Ca^2+^ activity serves to promote cellular and synaptic refinement. In this review, we summarize the current insights into the cellular and molecular mechanisms responsible for generating and modulating these spontaneous Ca^2+^ signals in the developing cochlea, and how they regulate the activation of auditory afferent fibres.

## Introduction

**Fig. 1:**
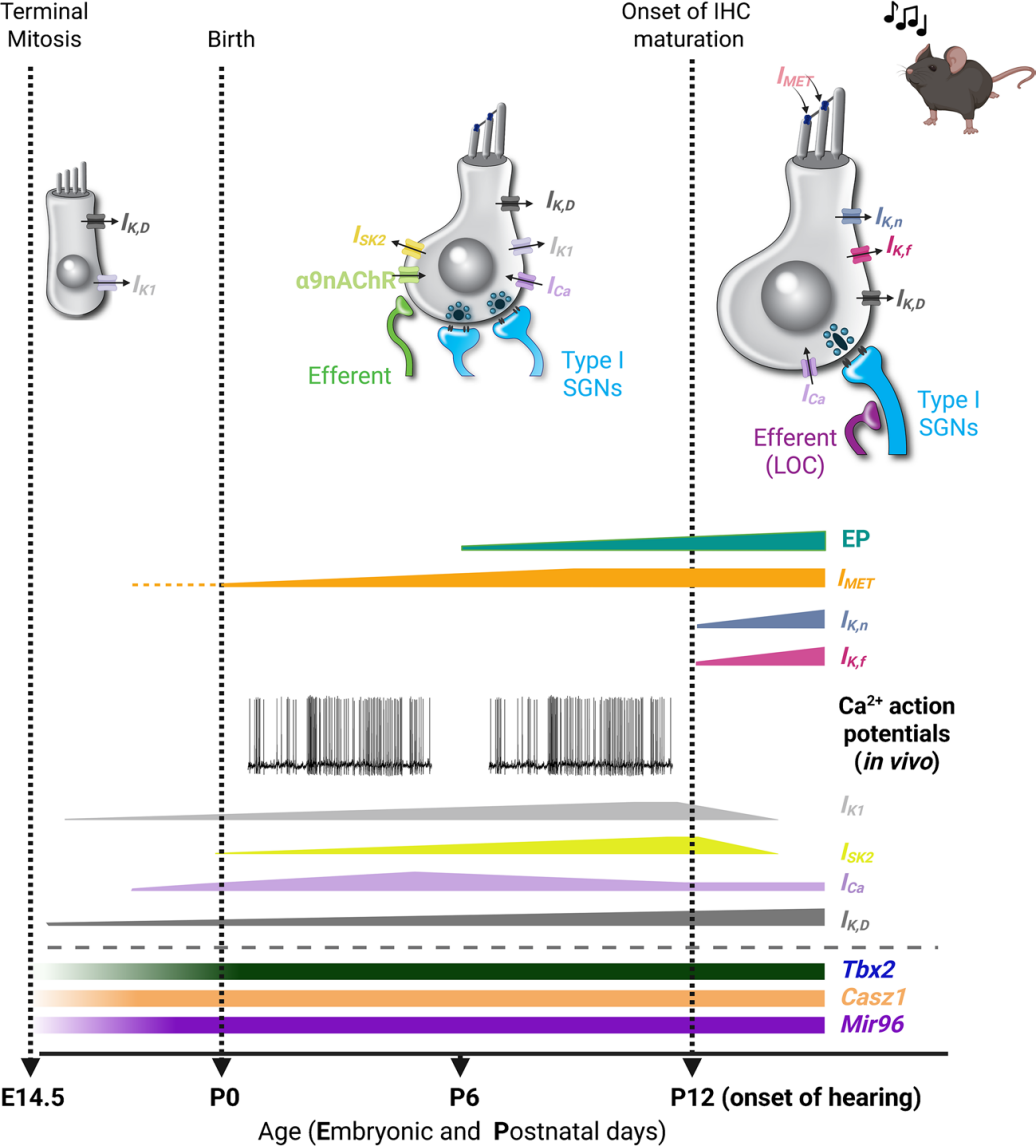
Functional maturation of IHCs from the apical coil of the mouse cochlea. Diagram depicting some of the biophysical and morphological changes during IHC development. Note that similar changes occur in the basal cochlea, but with the onset shifted a few days earlier. The day of birth (P0) corresponds to E19.5 [4]. The maturation of IHCs into functional auditory receptors begins around P12. The shaded bars (bottom) represent the temporal expression of some of the known genes involved in IHC functional differentiation (*Tbx2*, *Casz1* and *Mir96*) [5–7]. The height of the horizontal bars indicates relative changes in the size of the different basolateral membrane currents, the mechanoelectrical transducer current (*I*_*MET*_), and endocochlear potential (EP). During embryonic development, IHC total basolateral current is dominated by the inward rectifier (*I*_K1_) and delayed-rectifier (*I*_K,D_) K^+^ currents. At this stage, afferent and efferent fibres extend within the sensory epithelium, but do not contact IHCs yet. Just before birth, IHCs begin to show a small Ca^2+^ current (*I*_*Ca*_), which drives spontaneous Ca^2+^-dependent action potentials (evidence from ex vivo work: [4, 8]). In vivo recordings showed that IHCs generate spontaneous Ca^2+^-dependent transients (i.e. action potentials) throughout the pre-hearing stages of development [9, 10]. Postnatally, IHCs are transiently innervated by the efferent system, which includes the post-synaptic channels carrying the small conductance Ca^2+^-activated K^+^ current (*I*_SK2_) and a current mediated by α9α10 nicotinic acetylcholine receptors (nAChRs) [11–14]. The mechanoelectrical transducer current (*I*_MET_) appears at or just after birth. Around P0, both afferent and the transient efferent fibres establish synaptic contact with IHCs. During this time, type I SGN afferent fibres undergo pruning. Adult-type basolateral membrane currents (large conductance Ca^2+^-activated *I*_K,f_, and the negatively activating *I*_K,n_) appear from around P12. Type I SGNs form one-to-one connections with IHCs, and the efferent system (LOC) forms synapses on the afferent terminals

**Fig. 2:**
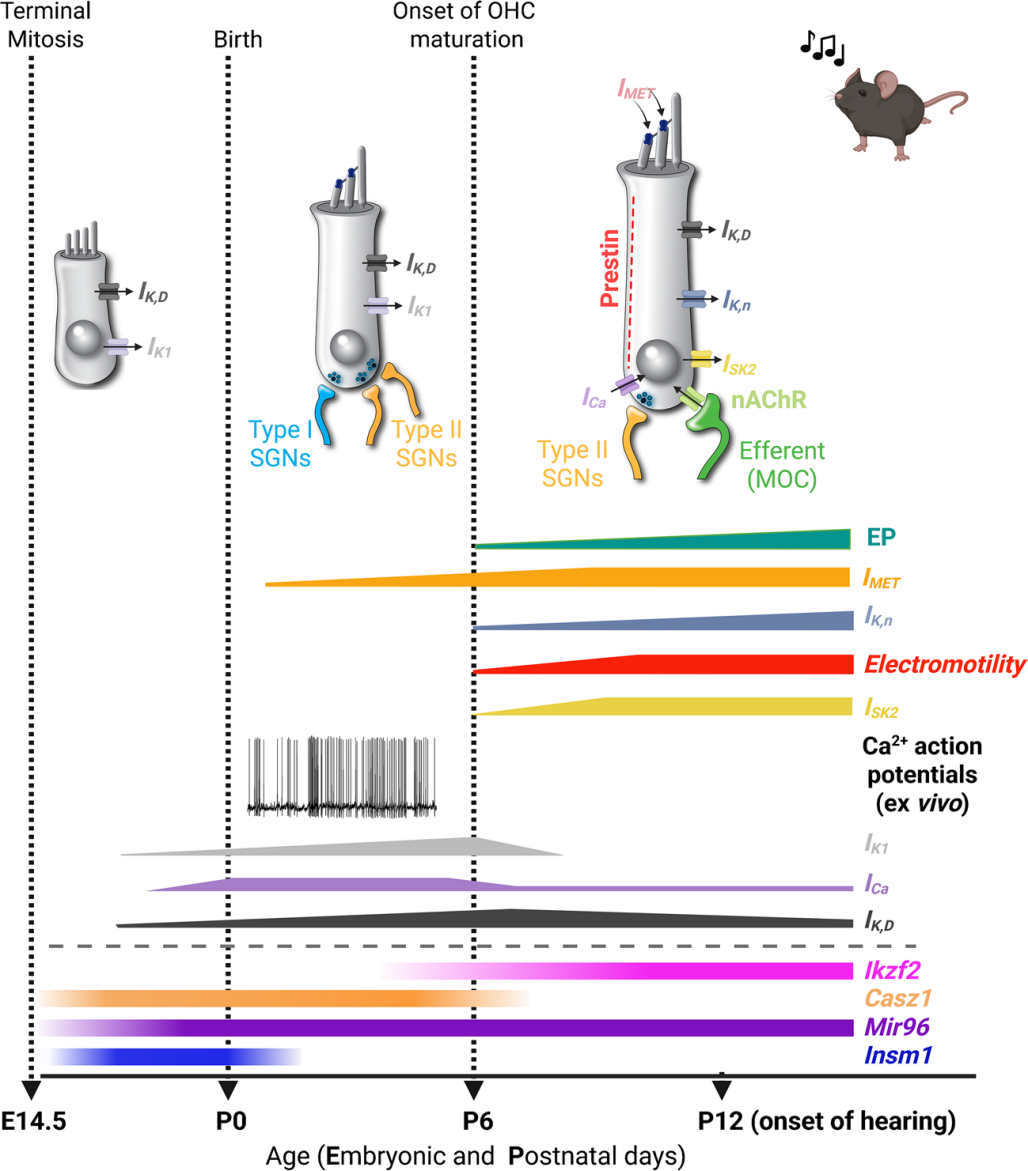
Functional maturation of apical-coil OHCs of the mouse cochlea. This diagram follows the format of Fig. 1 but depicts the development of OHCs. The maturation of OHCs into functional auditory receptors begins around P6-P8. Shaded bars (bottom): expression of some of the known genes involved in OHC functional differentiation (*Insm1*, *Casz1*, *Mir96* and *Ikzf2*) [5, 7, 15]. During embryonic development, OHCs primarily exhibit the inward rectifier (*I*_K1_) and delayed rectifier (*I*_K,D_) K^+^ currents. At this stage, afferent and efferent fibres extend within the sensory epithelium, but do not contact OHCs yet. Just before birth, OHCs begin to show a small Ca^2+^ current (*I*_*Ca*_). Unlike IHCs (Fig. 1), early postnatal OHCs do not exhibit additional basolateral membrane currents; however, the increased size of *I*_*Ca*_ likely drives spontaneous Ca^2+^-dependent transients (i.e. action potentials) for a brief period (from ex vivo work: [16]). *I*_MET_ appears starting around P1-P2. During this period, OHCs are contacted by both type I and type II SGNs. By the end of the first postnatal week, type II SGNs undergo pruning, and type I fibres are eliminated. The onset of functional maturation in OHCs is marked by the appearance of electromotile activity, which is driven by the motor-protein prestin located along the OHC lateral wall [17]. *Ikzf2* (see above), which encodes Helios, has been shown to be required for prestin expression. At the same time, the negatively activating delayed rectified K^+^ current *I*_K,n_ [18], is upregulated in maturing OHCs. Concurrent with the above basolateral changes, OHCs upregulate the efferent post-synaptic machinery, which includes the small conductance Ca^2+^-activated K^+^ current (*I*_SK2_) and α9α10 nicotinic acetylcholine receptors (nAChRs). In the mature configuration, OHCs are innervated by both the efferent MOC system and type II SGNs

The mammalian auditory system is finely tuned to detect acoustic stimuli with remarkable precision and sensitivity. This capability emerges from the integrated function of specialized morphological structures, such as the basilar and tectorial membranes [1, 2], and the different cell types within the cochlea [3]. The sensory epithelium of the mammalian cochlea, the organ of Corti, contains a highly organized array of specialized sensory hair cells (inner and outer hair cells: IHCs and OHCs: Figs. 1,2) and glia-like supporting cells.

The IHCs serve as the primary sensory receptors, transmitting acoustic information to the central auditory pathway via type I afferent fibres [19], which represent the majority of the spiral ganglion neurons (SGNs) innervating the cochlea (∼95 %) [20]. In the adult cochlea, type I SGNs form one-to-one axosomatic contact with a given IHC [21], and are directly modulated by the lateral olivocochlear (LOC) efferent system (Fig. 1) [22]. Unlike IHCs, the role of OHCs is to enhance cochlear sensitivity and frequency selectivity through somatic electromotility [23]. Adult OHCs are directly innervated by the medial olivocochlear (MOC) cholinergic efferent neurons (Fig. 2), which reduce cochlear amplification [22, 24]. OHCs are also sparsely contacted by type II afferent fibres (Fig. 2), constituting the remaining ∼5 % of SGNs. These type II SGNs form extensive spiralling arborizations with numerous OHCs and are thought to be activated by noxious sound stimulations [25–27], possibly through ATP-gated P2x7 receptors [28]. The cochlear epithelium also contains a diverse population of non-sensory supporting cells that maintain the structural and functional integrity of the organ of Corti [29, 30]. Beyond their roles in the adult cochlea, both sensory and supporting cells have been shown to regulate the functional maturation of the developing cochlea and the central auditory pathway prior to hearing onset.

As in other sensory systems [31], while the initial wiring of the auditory pathway depends on axon guidance molecules [32], circuit refinement is driven by spontaneous, patterned electrical activity that arises before the onset of external sensory input [33–35]. Indeed, neurons along the auditory pathway exhibit periodic bursts of spontaneous action potentials prior to hearing onset [36–38]. Multiple lines of evidence indicate that this spontaneous activity originates in the immature cochlea, as ablation of the sensory organ silences auditory neurons and disrupts their normal maturation [36, 39, 40]. This sensory-independent firing activity in the pre-hearing cochlea has been shown to be Ca^2+^-dependent and is required not only for glutamate release from hair cell ribbon synapses onto afferent terminals but also for regulating cochlear development. In this review, we summarize the current understanding of the origin and modulation of spontaneous Ca^2+^-dependent activity in the cochlea and discuss its role in establishing the mature sensory epithelium.

## Functional Maturation of IHCs and OHCs

During embryonic stages, the development of the mammalian cochlea is guided by intrinsic genetic programmes [41, 42]. In mice, terminal mitosis begins at the cochlear base around embryonic day 12 (E12) and progresses towards the apex over the following few days [43]. Since the onset of hearing occurs around postnatal day 12 (P12) in most altricial rodents, pro-sensory cells have approximately three weeks to functionally differentiate into sensory receptors capable of processing acoustic information (Figs. 1, 2). Within this developmental window, a combination of genetically programmed and physiologically driven mechanisms is required to generate functionally mature auditory receptors [41, 42, 44].

The initial differentiation between IHCs and OHCs is determined during embryonic stages by several transcription factors (INSM1, CASZ1, and TBX2) and the non-coding microRNA 96, which play a key role in consolidating the identity of both cell types (Figs. 1, 2) (recently reviewed by [45]). The final stage of hair cell maturation occurs postnatally with the acquisition of adult-like biophysical and morphological characteristics. The onset of IHC functional maturation, which occurs at about P10-P12, is marked by the appearance of the K^+^ current *I*_K,n_ and the fast-activating BK current *I*_K,f_ (Fig. 1) [4, 46, 47]. IHC maturation also coincides with the progressive removal of their efferent innervation and the downregulation of the IHC post-synaptic components (Fig. 1) (recently reviewed by [45]). Immature IHCs are also innervated by highly branched type I SGNs via ribbon synapses. Synaptogenesis in IHCs mainly occurs during postnatal stages of development, through a range of structural and functional refinements [19, 48], including the assembly of the pre-synaptic active zones [49, 50], which eventually establish the characteristic and highly specialized monosynaptic axosomatic contact (Fig. 1). In OHCs, the onset of functional maturation at about P6-P8 is characterized by the appearance of electromotile activity and, similar to IHCs, the expression of *I*_K,n_ (Fig. 2) (recently reviewed by [45]). While OHC somatic electromotility drives sound amplification within the cochlear partitions [51, 52], *I*_K,n_ is crucial for setting the resting membrane potential [18], a role that in immature OHCs is fulfilled by the delayed rectifier (*I*_K,D_) and the inward rectifier (*I*_K1_) K^+^ currents (Fig. 2) [18, 53]. Concurrently, the MOC efferent system establishes synaptic contacts with OHCs, a process that is accompanied by the expression of SK2 channels and α9α10-nAChRs at the postsynaptic basolateral membrane [22] (Fig. 2). The refinement of immature afferent innervation leads to a characteristic pattern consisting of type II fibres contacting several OHCs [25, 26].

## Calcium Signals in Developing Cochlear Inner Hair Cells

**Fig. 3:**
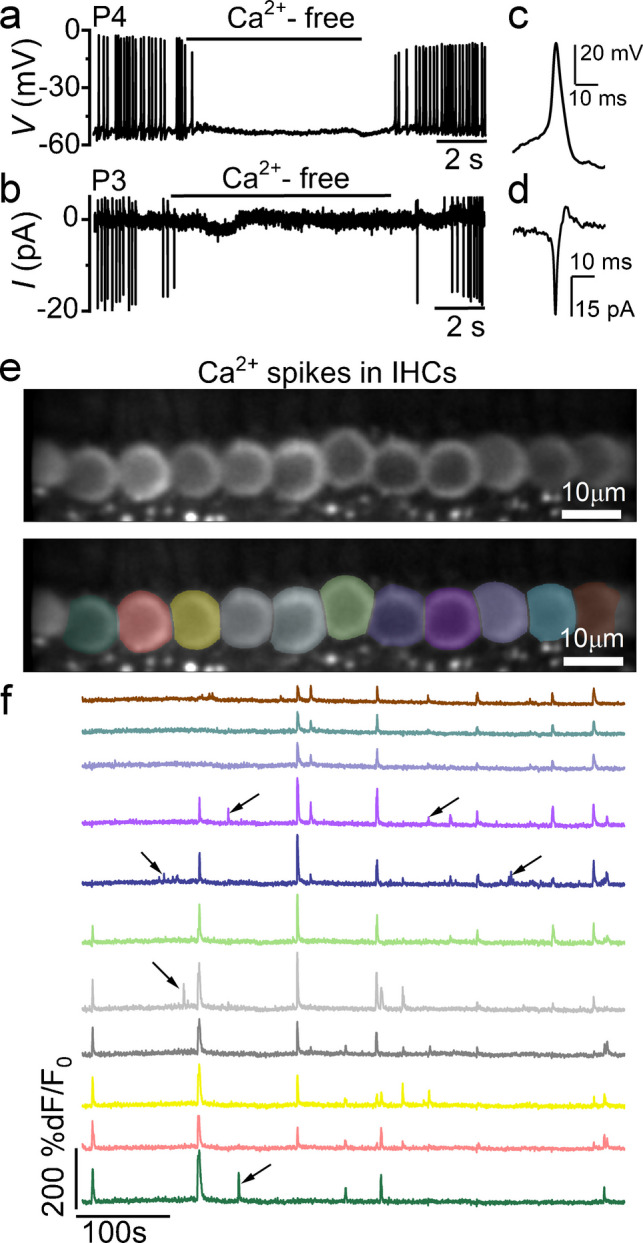
Spontaneous action potentials in pre-hearing IHCs. (**a**,**b**) Spontaneous action potentials recorded from an IHC in a cochlear explant using whole-cell current clamp (**a**) and cell-attached voltage clamp (**b**). Recordings were performed at body temperature in the presence of 1.3 mM extracellular Ca^2+^. Note that action potentials are reversibly abolished in Ca^2+^-free extracellular solution. (**c**,**d**) Single action potentials from panels (**a**) and (**b**), respectively, shown on an expanded time scale. Image modified from [57]. (**e**) Top: average intensity projection of a timelapse recording showing GCaMP6f expression in apical-coil IHCs from a live *GCaMP6*^*fl/fl*^*Myo15-Cre*^*±*^ mouse (P6). Bottom: Regions of interest (ROIs) generated using a semi-automated identification approach. These ROIs were used to measure spontaneous Ca^2+^ signals from individual IHCs shown in the top panel. (**f**) Fluorescence time series computed as pixel-averages from the ROIs in panel (**e**), demonstrating spontaneous Ca^2+^ activity in IHCs in vivo (colours match those in panel (**e**)). Image modified from [9]

Spontaneous activity in the developing cochlea has been extensively studied in mice and rats using *ex vivo* explants of the sensory epithelium. These pioneering studies showed that IHCs generate spontaneous and evoked Ca^2+^-dependent action potentials prior to hearing onset (Fig. 1) [8, 46, 54–56]. These action potentials are elicited by an inward Ca^2+^ current flowing through Ca_V_1.3 channels (*I*_Ca_: Fig. 1 and Fig. 3a-d), which account for >90 % of the total Ca^2+^ current in IHCs [58]. Although pre-hearing IHCs also express a TTX-sensitive Na^+^ current, this is not required for initiating action potentials but, instead, it modulates their frequency by shortening the time to reach threshold [8]. Action potential repolarization is mainly driven by a delayed rectifier K^+^ current (*I*_K,D_) [4, 46], an inward rectifier K^+^ current (*I*_K1_) [53] and a transiently expressed small conductance Ca^2+^-activated K^+^ current (*I*_SK2_) [11–13] (Fig. 1).

Although *ex vivo* experiments have been invaluable for elucidating the biophysics underpinning Ca^2+^ action potentials in developing IHCs, they have been less effective in identifying the cellular mechanisms responsible for initiating and modulating this activity under *in vivo* conditions. For example, early studies showed that IHCs generate spontaneous action potentials only up to the end of the first postnatal week (Fig. 1). From the second postnatal week until hearing onset, IHCs maintained in cochlear explants undergo a progressive hyperpolarization of their resting membrane potential, reaching values more negative than the activation threshold of *I*_Ca_ (more negative than −65 mV) [59]. This shift, which is driven by the developmental increase in the size of *I*_K1_ (Fig. 1) [53], prevents spontaneous Ca^2+^ spiking. Consequently, in cochlear explants from mice aged P7-P12, action potentials could only be evoked by artificial depolarization of IHCs [4, 57, 60]. However, additional *ex vivo *work has shown that IHCs are intrinsically silent throughout pre-hearing development. Instead, Ca^2+^ action potentials in IHCs are driven by spontaneous ATP release from supporting cells [61, 62].

The origin of these Ca^2+^ action potentials has recently been addressed using an* in vivo* experimental approach that allowed the measurements of spontaneous Ca^2+^ signals in the developing cochlea in anaesthetised live mice expressing GCaMP in IHCs [9, 10]. This in vivo study demonstrated that IHCs can generate sporadic spontaneous Ca^2+^ transients (Fig. 3e,f) throughout pre-hearing stages of development, independently of supporting cell activation (Fig. 1) [62]. The discrepancy between* ex vivo* and *in vivo* conditions is likely due to the absence in cochlear explants of the distinct ionic compositions of the endolymph and perilymph [63], and consequently the lack of the endocochlear potential [64], which starts to appear from around the end of the first postnatal week (Fig. 1). The relatively low-Ca^2+^ concentration in the endolymph (~300 μM in prehearing mice) [60], combined with the developing endocochlear potential (~15–40 mV, P6-P10) [9, 64], likely promotes the opening of MET channels that are already functional during pre-hearing stages. This drives an inward depolarizing MET current into the immature IHCs (*I*_*MET*_: Fig. 1), which contributes to setting the resting membrane potential of pre-hearing IHCs near the activation threshold of Ca^2+^ channels [59], thereby promoting spontaneous action potential activity. Furthermore, unlike cochlear explants, the *in vivo* approach preserves another critical aspect of cochlear IHC function, which is the inhibitory efferent cholinergic input originating in the brainstem, which transiently innervates IHCs during immature stages of development [11–14].

## Spontaneous Activity in Developing OHCs

**Fig. 4:**
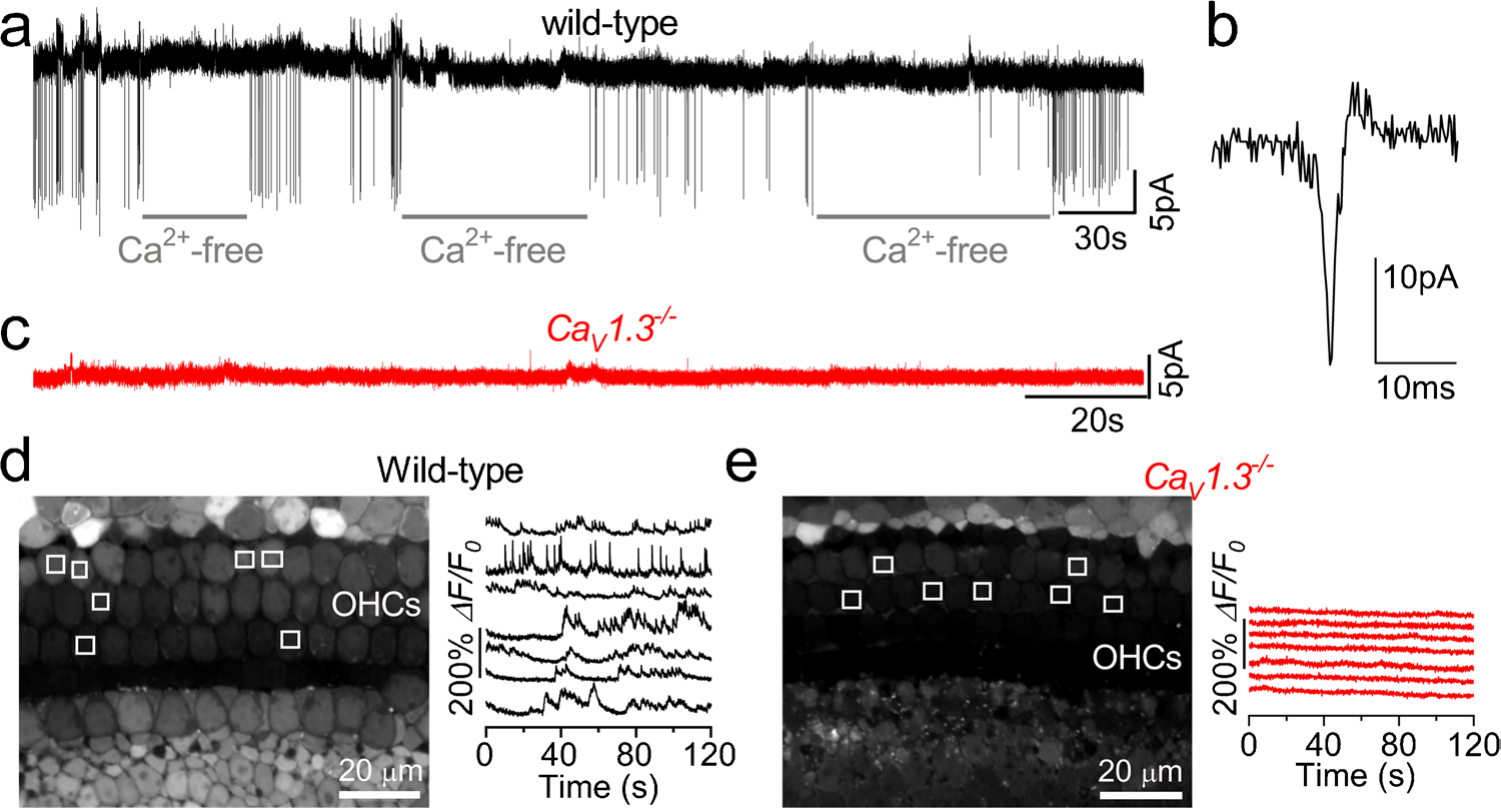
Spontaneous action potentials in immature OHCs. (**a**) Spontaneous currents recorded from a P2 mouse OHC using cell-attached voltage clamp at body temperature with 1.3 mM extracellular Ca^2+^. Note that the local perfusion of a Ca^2+^-free extracellular solution reversibly abolishes the currents. (**b**) Expanded view of a single current transient from panel (**a**). (**c**) Cell-attached recordings from an OHC of a P1 *Ca*_*V*_*1.3* knockout mouse (*Ca*_V_*1.3*^*−/−*^), highlighting the dependence of spontaneous activity on Ca^2+^ channels. (**d**, **e**) Representative *ΔF/F*_*0*_ traces from OHCs of a P2 wild-type (**d**) and a P1 *Ca*_*V*_*1.3*^*−/−*^ (**e**) mouse. Traces are computed as pixel averages of regions of interest (white squares) centred on OHCs. Image modified from [66]

In the mouse cochlea, the onset of OHC functional maturation occurs at around P6-P7, nearly a week earlier than IHCs (Fig. 2). However, during the first postnatal week, whole-cell patch-clamp recordings from OHCs in cochlear explants have shown that action potentials can be elicited either by membrane depolarization [18] or by using highly elevated extracellular Ca^2+^ concentrations (10 mM) [65]. From P7 onwards, the presence of the outward K^+^ current *I*_k.n _(Fig. 2), which is largely active at the resting membrane potential [18], likely prevents spiking activity in OHCs. Some evidence for the possible presence of spontaneous Ca^2+^ action potentials in OHCs during the first 3–4 postnatal days comes from two-photon imaging or cell-attached patch clamp experiments (Fig. 4) [16, 66]. These methods better preserve the intracellular milieu, including endogenous Ca^2+^ buffers, compared to the whole-cell approach. However, it remains unknown whether immature OHCs, like IHCs, can fire spontaneous Ca^2+^ action potentials *in vivo* and, if so, whether this activity is restricted to just a few days, as observed in cochlear explants.

## Purinergic Signalling in the Supporting Cells of the Developing Cochlea

**Fig. 5:**
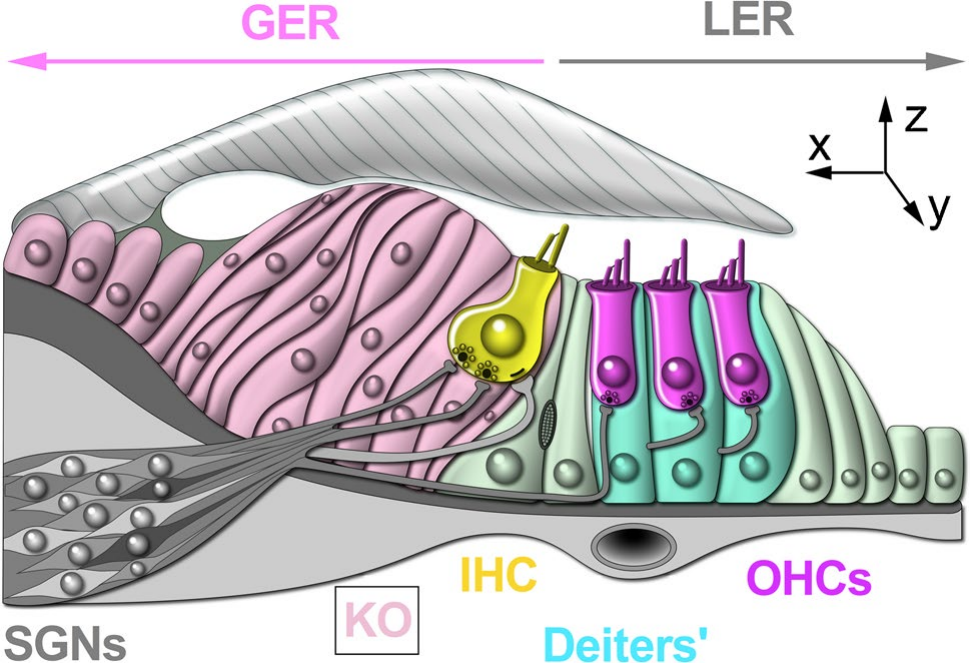
Diagram of the pre-hearing sensory epithelium of the cochlea. Diagram depicting a cross-section of an immature organ of Corti, illustrating the location of the sensory hair cells and supporting cells. Spontaneous Ca^2+^ waves are generated in the supporting cells present in the greater epithelial ridge (GER) but not in the lesser epithelial ridge (LER). Image modified from [16]

The sensory epithelium of the developing cochlea includes the greater epithelial ridge (GER) and the lesser epithelial ridge (LER) (Fig. 5), which become recognizable from around E16 [67]. The LER contains the OHCs and adjacent supporting cells in the lateral portion of the epithelium, whereas the GER includes IHCs and supporting cells located medially. During this developmental period, the GER contains Kölliker’s organ, a transient structure that plays a critical role in early development and maturation of the auditory system [68]. Similar to astrocytes in the central nervous system [69], the supporting cells of Kölliker’s organ actively generate complex Ca^2+^ signals that can propagate as intercellular Ca^2+^ waves throughout the sensory epithelium [16, 61, 70–72]. In the cochlea, this spontaneous Ca^2+^ activity ceases around the onset of hearing in mice [9, 71]. This follows the regression of Kölliker’s organ, and the downregulation of several key molecules involved in the signalling cascade [62, 73, 74].

**Fig. 6:**
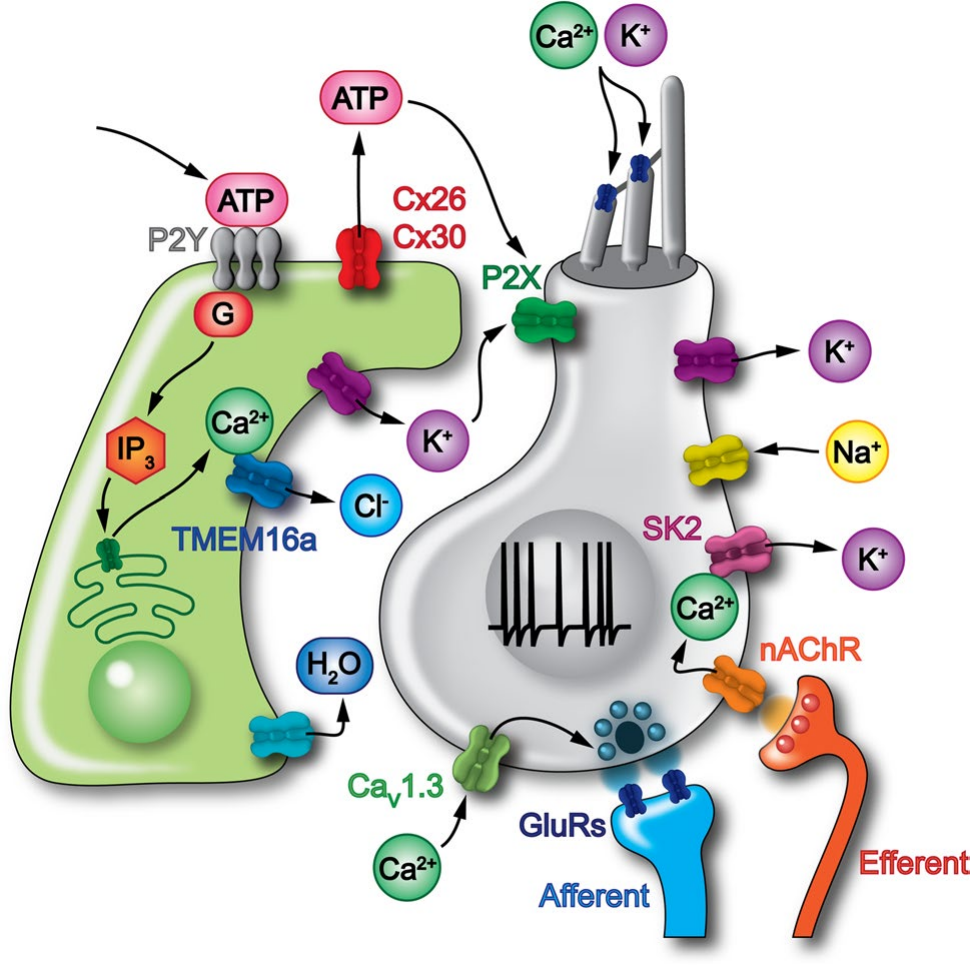
Supporting cell-IHC interaction in pre-hearing mice. Schematic illustrating the key molecular components and ionic currents in the supporting cells (left) and IHCs (right) required for their function. Supporting cells can release ATP into the extracellular space either spontaneously or in response to hair-cell stimulation [75], mechanical stress [76], or damage [77, 78]. Connexin hemichannels are the most likely candidates as the conduit, however different mechanisms have been proposed [79, 80]. This localized release of ATP likely initiates the intercellular cascade that drives the propagation of Ca^2+^ waves in supporting cells through paracrine activation of G protein-coupled P2Y1 receptors [71, 81]. ATP-induced Ca^2+^ increase opens TMEM16A Ca^2+^-activated Cl^−^ channels, driving Cl^−^ efflux followed by water and K^+^ efflux to maintain osmotic balance and electroneutrality [62]. Accumulation of K^+^ in the intercellular space between supporting cells and IHCs has been proposed to increase the frequency of spontaneous action potentials in IHCs and to coordinate the firing of several adjacent IHCs (for more details see main text). In IHCs, currents are as described in Fig. 1. IHCs are innervated by type I afferent fibres, which release glutamate onto post-synaptic glutamate receptors (GluRs). Pre-hearing IHCs are also transiently innervated by the efferent fibres, which release acetylcholine (ACh) onto pre-synaptic α9α10 nicotinic acetylcholine receptors (nAChRs). Supporting cells express IP_3_ receptor (IP_3_R), transmembrane protein 16 A (TMEM16A, also known as Anoctamin-1, ANO1), Ca^2+^-activated Cl^−^ channels, connexin 26 (Cx26), connexin 30 (Cx30) and G-coupled metabotropic purinergic receptors (P2YR)

Pharmacological studies have shown that these Ca^2+^ waves are mediated by the binding of extracellular ATP, which is released by the supporting cells in the GER, to G protein-coupled P2Y autoreceptors located on the endolymphatic surface of supporting cells. P2Y receptor activation triggers the phospholipase-C dependent generation of IP_3_, which binds to its receptors on the ER, raising the concentration of cytosolic free Ca^2+^ and triggering additional ATP release (Fig. 6). The intercellular diffusion of IP_3_ through gap-junction channels (connexin 26 and 30) enables the propagation of Ca^2+^ signals as an intercellular wave (recently reviewed by [45]). Interestingly, there is no evidence of spontaneous Ca^2+^ waves originating in the LER, even though supporting cells in both GER and LER share similar Ca^2+^ signalling cascade [82].

## Spontaneous Ca^2+^ Waves Regulate IHC Firing Activity in the Prehearing Cochlea

Spontaneous ATP release from supporting cells into the extracellular space of the GER (Kölliker’s organ) initiates the signalling cascade that drives the generation and propagation of discrete Ca^2+^ waves within the sensory epithelium. Although this mechanism has been observed using Ca^2+^ imaging in both cochlear explants and *in vivo*, the dynamics of these Ca^2+^ waves differ between the two conditions. In cochlear explants, supporting cells generate Ca^2+^ waves that are significantly larger and slower [9, 56, 61, 83] than those recorded *in vivo* [9, 10]. Given that supporting cells release ATP in response to cochlear damage [77, 78], it is possible that the large Ca^2+^ waves in explants are, at least partially, a direct consequence of tissue dissection.

Both *ex vivo* and* in vivo* studies have shown that spontaneous Ca^2+^ waves occurring near IHCs can depolarize them, leading to coordinated bursts of Ca^2+^ action potentials across several neighbouring cells [9, 61, 70, 81]. *Ex vivo* studies showed that ATP-induced activation of P_2_Y_1_ receptors in supporting cells leads to elevated intracellular Ca^2+^ and the opening of TMEM16A, which ultimately cause K^+^ efflux in the extracellular space (Fig. 6). Elevated extracellular K^+^ depolarizes adjacent IHCs, triggering their synchronous activity. Subsequently, the osmotic shrinkage of supporting cells expands the extracellular volume, diluting K^+^ concentration and terminating IHC depolarization [81]. However, while the role of TMEM16A in synchronizing IHC activity is consistent across *ex vivo* studies, its direct involvement in generating Ca^2+^ waves is less clear, as conflicting evidence exists [62, 72].

**Fig. 7:**
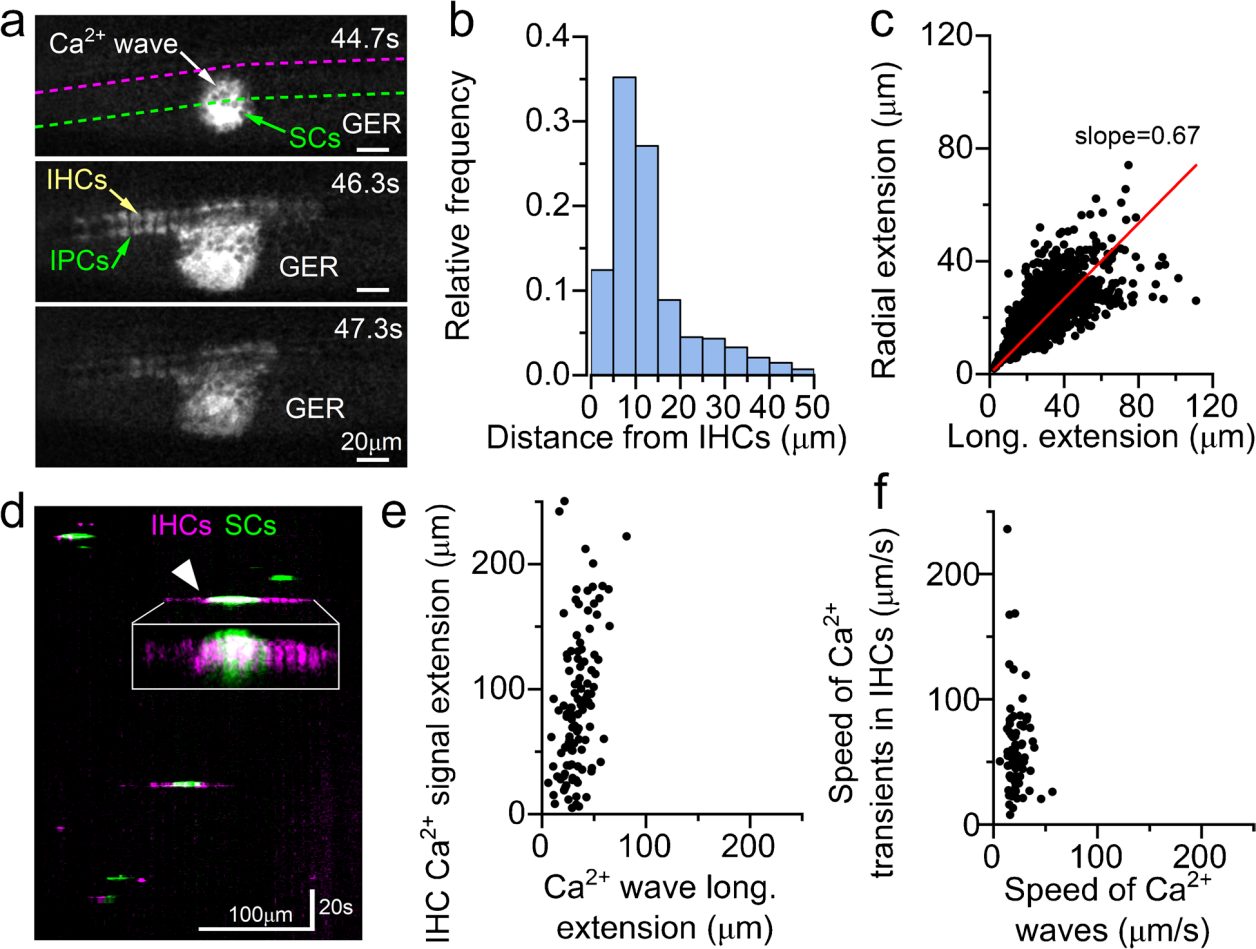
Spontaneous Ca^2+^ signals in supporting cells and IHCs in pre-hearing live mice. (**a**) Representative images showing spontaneous Ca^2+^ waves propagating from supporting cells in the GER towards the IHCs of a live P4 *GCaMP6f*^*l/fl*^*Pax2-Cre*^*±*^ mouse. SCs: supporting cells within the GER; IPCs: inner phalangeal cells located in between the IHCs. (**b**) Histogram displaying the number of Ca^2+^ waves as a function of their distance relative to the IHCs. (**c**) Maximal longitudinal extension of Ca^2+^ waves from panel (**b**) plotted as a function of their radial extension. (**d**) Kymograph constructed by drawing lines across the IHCs (magenta) and GER (green) regions from panel (**a**). Inset: magnification of the Ca^2+^ wave indicated by the arrowhead, highlighting the larger longitudinal spread of the Ca^2+^ signal in the IHCs compared to that of the GER. (**e**, **f**) Relationship between the spread (**e**) and speed (**f**) of Ca^2+^ waves in the supporting cells of the GER and Ca^2+^ signals in IHCs, demonstrating the faster and wider extension of Ca^2+^ signals in the latter. Image modified from [9]

In vivo recordings have confirmed this complex interaction between IHCs and supporting cells, showing that spontaneous Ca^2+^ waves are essential for synchronizing the firing activity of nearby IHCs. Yet, the long-range longitudinal propagation of Ca^2+^ signals within IHCs appears to occur independently of Ca^2+^ wave propagation through supporting cells (Fig. 7) [9]. The mechanism driving the long-range longitudinal propagation of Ca^2+^ signals in IHCs [9], which occurs more rapidly than Ca^2+^ wave propagation [71], remains unknow. It is possible that during sustained firing, K^+^ efflux from active IHCs accumulates in the intercellular space, depolarizing neighbouring IHCs and thereby directly triggering their activation.

**Fig. 8:**
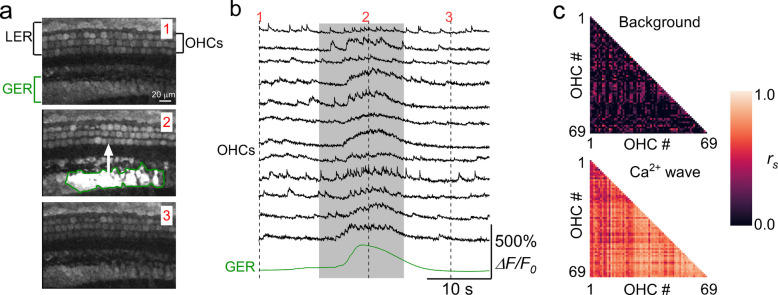
Calcium waves and OHC Ca^2+^ signalling from cochlear explants. (**a**) Three representative images (left panels) obtained before (1), during (2) and after (3) the spontaneous appearance of a large Ca^2+^ wave in the GER in the apical coil of a P2 wild-type mouse. (**b**) Representative ΔF/F_0_ traces from 12 OHCs (black traces) and that originating from the Ca^2+^ wave in the GER (green traces). The grey-shaded area highlights the time window used for correlation analysis. Recordings were made at 31 °C. (**c**) Correlation matrices computed from the Ca^2+^ fluorescence traces of 69 OHCs. Correlation coefficients were computed before (top panels: background) and during (bottom panels: Ca^2+^ wave) the occurrence of the Ca^2+^ wave in nearby supporting cells. Each matrix element represents the Spearman’s rank correlation coefficient of one pair of OHCs. Image modified from [16]

Much less is known about the regulation of Ca^2+^ signals in the LER. Although the LER itself does not appear to generate spontaneous Ca^2+^ waves (see above), recent studies in cochlear explants and organotypic cultures have shown that waves originating in Kölliker’s organ can propagate into supporting cells of the LER (Fig. 8) [16, 84]. These GER-derived Ca^2+^ waves induce Ca^2+^ elevation in Deiters’ cells, which lead to synchronized Ca^2+^ activity in OHCs of neonatal mice [16]. Whether this mechanism also operates under *in vivo* conditions remains unknown. Consistent with the absence of spontaneous Ca^2+^ waves in the LER, ATP release from supporting cells in this region has been reported primarily following exposure to noxious stimuli [77, 78], or in response to the extracellular application of ATP to hair cells [75, 85].

## Spontaneous Activity in Type I SGNs

**Fig. 9:**
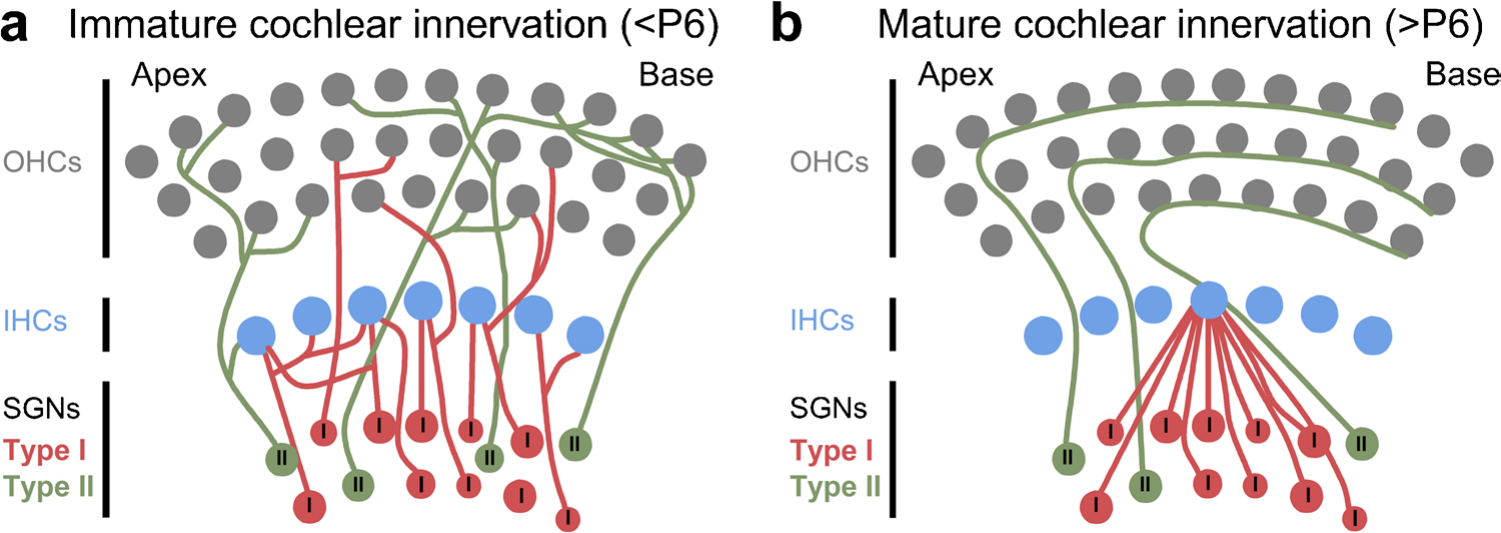
Diagram illustrating the developmental refinement of SGN afferent terminals in the pre-hearing sensory epithelium. Diagram showing the innervation pattern targeting IHCs and OHCs during the first (**a**) and second (**b**) postnatal week of mouse cochlear development. Following extensive pruning of SGN afferent fibres, the mature configuration is established. This consists of: Type I SGN fibres (~95 %), which are unbranched, myelinated neurons that connect with a single IHC (approximately 10–20 neurons per IHC); and Type II SGN fibres (the remaining ~5 %), which are unmyelinated neurons that spiral towards the base of the cochlea to contact multiple OHCs

During pre-hearing stages of development, IHCs are innervated by both the afferent and efferent systems (Fig. 1) [19, 22]. Type I SGN afferent fibres form axo-somatic contacts with IHCs shortly before birth around E16 in the basal turn, extending apically by E17-E18 [48]. At this age, IHCs generate broad, immature Ca^2+^ action potentials, that nevertheless trigger the fusion of glutamate-containing vesicles at their ribbon synapses [8], driving action potential activity in SGNs as early as P0 [86]. Before the onset of hearing, immature IHCs are innervated by highly branched type I SGNs, which eventually establish the characteristic one-to-one axosomatic contact following extensive pruning (Fig. 9) [19, 48, 87]. This loss of SGN terminals is accompanied by a corresponding decrease in the number of presynaptic ribbons [88, 89].

**Fig. 10:**
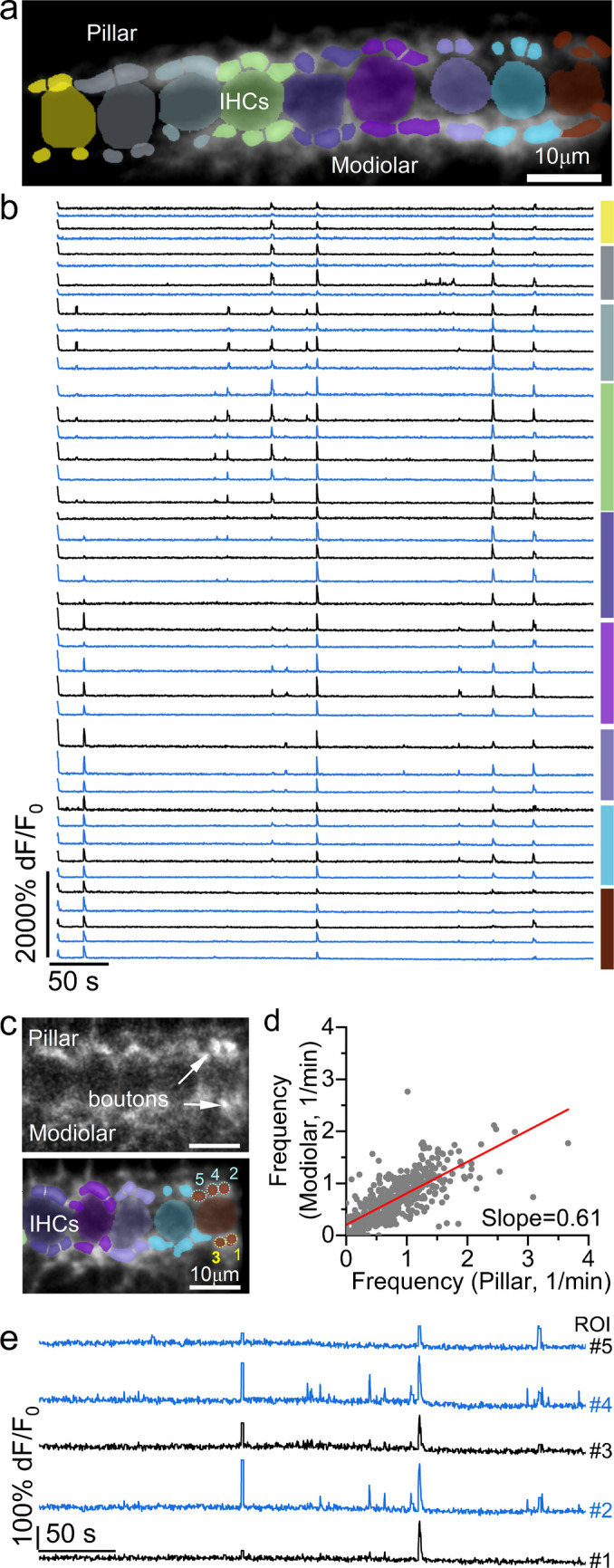
Spontaneous Ca^2+^ signals in postsynaptic afferent terminals from pre-hearing live mice. (**a**) Average intensity projections displaying GCaMP6f expression in vivo from a P4 *GCaMP6f*^*l/fl*^*NeuroD-Cre*^*±*^ mouse. Image shows superimposed segmentation mask highlighting ROIs for identified synaptic terminals colour-matched to their associated IHC body. (**b**) Fluorescence traces of pillar (blue) and modiolar (black) afferent terminals identified by the ROIs shown in panel (**a**). Lines on the right of the traces indicate the terminals belonging to the colour-matched IHC in panel (**a**). While single IHC Ca^2+^ events show scattered activity in individual afferent terminals, coordinated Ca^2+^ events across multiple IHCs consistently recruit a large number of terminals. (**c**) Average intensity projection displaying GCaMP6 signal in vivo from a P4 *GCaMP6f*^*l/fl*^*NeuroD-Cre*^*+*^ mouse. Bottom panel: ROIs highlighting individual afferent terminals assigned to the IHCs in the bottom panel. (**d**) Relationship between the frequency of Ca^2+^ transients in the afferent terminals positioned in the modiolar and pillar side of individual IHCs. (**e**) Fluorescence traces of the pillar (blue) and modiolar (black) ROIs numbered 1–5 in panel (**c**). Image modified from [9]

Ex vivo patch-clamp recordings from individual SGN terminals, pioneered by Glowatzki and colleagues (e.g., [90, 91]), have been instrumental in uncovering how IHC glutamate release shapes SGN responses [19, 92]. However, how the diverse Ca^2+^ dynamics in IHCs are faithfully transmitted to SGN terminals in the intact developing cochlea remains largely unknown. Ex vivo imaging studies in P7-P9 mice have reported sparse, long-lasting Ca^2+^ transients in SGN terminals, with little correlated activity across terminals contacting the same or neighbouring middle-apical IHCs [9]. This is surprising, given that IHCs appear capable of driving Ca^2+^-induced glutamate release at ribbon synapses under similar experimental conditions (see above). In contrast, in vivo experiments have shown that SGN terminals display rapid and highly correlated Ca^2+^ transients that closely match IHC activity (Fig. 10) [9]. Furthermore, the synchronization of IHCs by Ca^2+^ waves in the supporting cells has been shown to increase the number of SGN terminals activated by a single IHC in live mice [9]. This is likely to reinforce the refinement of neuronal projections to discrete areas of the developing auditory pathway [33, 35].

In the adult mammalian cochlea, type I SGNs exhibit a wide range of spontaneous rate (SR) activity, with low-SR neurons typically having higher activation thresholds compared to those with high SR [93–96]. SGN subtypes segregate around the IHC synaptic region, with low-SR fibres contacting the modiolar side (facing the central axis of the cochlea), while high-SR fibres contact their pillar side (facing the OHCs) [94, 97]. This functional diversity is likely required to convey the wide dynamic range of sound intensity encoded by each IHC [98]. In mice, single-cell RNA sequencing studies have identified three SGN subtypes based on unique molecular marker combinations, the identity of which is primarily defined during pre-hearing stages of development (e.g., [96, 99–101]). However, electrophysiological measurements in adult mice did not reveal three distinct functional subclasses with strict spatial segregation around IHCs [96]. Instead, the authors identified a group of *Lypd1*-expressing SGNs with low SRs that preferentially innervate the IHC modiolar side, and a second *Calb2*-positive population exhibiting a wider range of SRs that preferentially innervates the IHC pillar side [96]. Although the bimodal distribution in type I afferent SRs identified in cats is less evident in mice, some degree of spatial segregation appears to exist in both in adult [96] and pre-hearing mice [102]. In vivo recordings have also revealed that the frequency and amplitude of Ca^2+^ transients are higher in SGN terminals on the pillar side of the IHCs compared to those on the modiolar side (Fig. 10) [9]. This further supports the notion that, alongside molecular identity, the functional segregation of SGNs is likely established during pre-hearing stages of development.

In addition to the afferent system, IHCs form transient axo-dendritic synapses with the cholinergic efferent fibres, which originate in the brainstem [22]. Acetylcholine (ACh) released by efferent terminals activates α9α10 nicotinic ACh receptors (nAChRs) on IHCs, causing Ca^2+^ influx that, in turn, opens small-conductance Ca^2+^ activated K^+^ channels (SK2). The resulting K^+^ efflux hyperpolarizes IHCs, thereby reducing their excitability (Fig. 6) (e.g., [11–13]). Ex vivo studies using artificial activation of the efferents have demonstrated that this system provides an inhibitory feedback mechanism capable of directly modulating spontaneous Ca^2+^ activity in developing IHCs. However, the exact physiological role of the efferent system in regulating IHC firing in vivo remains unknown.

## Refinement of Type II SGN Connections

Mature OHCs receive cholinergic input via medial olivocochlear (MOC) efferent neurons originating in the brainstem [22]. OHCs are also innervated by type II SGNs, which turn towards the base of the cochlea to form en-passant ribbon synaptic contacts with 5–30 OHCs (Fig. 9) [26, 27]. Each OHC receives two or three SGN terminals with juxtaposed ribbons (Fig. 9) [27, 103]. This mature morphological organization is established towards the end of the first postnatal week following extensive axon outgrowth, target selection, refinement, and pruning of SGN peripheral processes [25, 104]. During this refinement, approximately 25 % of the type II SGNs innervating OHCs are lost, while the type I SGN terminals that transiently innervate immature OHCs are eliminated (Fig. 9) [25, 105]. Although the role of type II SGNs remains largely unclear, they appear to respond to sound levels capable of causing cochlear damage, suggesting they function as cochlear nociceptors [106, 107]. This conclusion is derived from *ex vivo* electrophysiological recordings demonstrating that type II SGNs are only weakly depolarized by OHCs, suggesting that the synchronized activation of several OHCs connected to the same afferent fibre is likely required to reach the firing threshold [27, 106, 108]. Recent evidence indicates that type II SGNs can be directly modulated by MOC efferent terminals via the release of GABA, instead of the classical neurotransmitter ACh [109], the role of which could be to fine tuning the output of the OHCs.

## Role of Spontaneous Calcium Signalling in the Developing Cochlea

Spontaneous Ca^2+^ activity in the developing mammalian cochlea has been implicated in multiple processes, including the maturation of hair cells [70, 110–112], the survival of nascent neurons and the refinement of the neural circuitry (recently reviewed by [45, 113].

Disrupting spontaneous Ca^2+^-dependent signalling in the developing cochlea is known to affect the functional maturation of the sensory epithelium. For example, altering the temporal pattern of IHC Ca^2+^ action potentials during the second postnatal week of cochlear development impairs maturation of their synaptic machinery [110, 114]. Moreover, suppression of IHC spiking during the same period disrupts the normal morphological and functional maturation of their stereociliary bundles [112]. Furthermore, mice lacking the MET current, which normally drives depolarization in IHCs during the second postnatal week due to the establishment of the endocochlear potential (Fig. 1) [60, 64], fail to upregulate mature basolateral membrane currents (*I*_*K,n*_ and *I*_*K,f*_) [115, 116]. Together, these findings support the existence of a “critical period” in the second postnatal week, just prior to hearing onset, during which Ca^2+^ activity is essential for IHC maturation. Much less is known about OHCs, but recent work showed that abolishing their Ca^2+^-induced firing activity, which unlike in IHCs, is restricted to the first postnatal week, leads to dysregulation of type II SGN afferent connectivity [16].

A recent study has also demonstrated that the developing stereocilia of both IHCs and OHCs exhibit spontaneous Ca^2+^ transients [117]. Interestingly, these Ca^2+^ signals occur not only at the tip of transducing stereocilia, where the MET channel is located [118], but also along the stereocilia shaft and within the microvilli-like structures on the hair cell surface [117]. These Ca^2+^ transients are not triggered by the propagating Ca^2+^ waves from the GER, they are short-lived and unable to induce hair-cell depolarization. Although it is currently unknown whether these Ca^2+^ signals are present in the mouse cochlea *in vivo*, they could potentially be involved in regulating hair bundle development [119, 120].

Spontaneous cochlear activity is also critical for the refinement of the central auditory pathway. Peripheral activity propagates centrally along the auditory pathway [86, 121], since functional synapses are already established in the brainstem at birth [122], and neurons in the superior olivary complex can fire action potentials in pre-hearing rodents [123]. Cochlear ablation or block of spontaneous activity in the sensory epithelium abolishes firing in the auditory centres and leads to substantial loss of cochlear nucleus neurons. Furthermore, removing efferent feedback to IHCs disrupts tonotopic map refinement in the medial nucleus of the trapezoid body (MNTB) and impairs bilateral coupling in the inferior colliculi (recently reviewed by [45, 113]).

## Conclusions and Future Work

A combination of single-cell and systems neuroscience approaches, applied to both *in vivo* and *ex vivo* models, has considerably furthered our understanding of the mechanisms driving early spontaneous activity in the cochlea and its role in the maturation of the auditory system. The emerging picture suggests that the interactions between Ca^2+^ and electrical activity in the developing cochlea influence gene expression programs and the refinement of immature neural connections and sensory domains along the auditory pathway.

Nevertheless, several fundamental questions remain currently unaddressed. For example, what initiates ATP release from developing supporting cells, and what are the mechanisms underpinning it? How does the inhibitory efferent system modulate the spiking activity of developing IHCs, and what is the activity pattern of the efferent fibres *in vivo*? Does glutamate release dynamics under in vivo conditions mirror findings obtained using *ex-vivo* preparations? Furthermore, there is evidence showing that action potentials occurring in IHCs during the second postnatal week are required for their correct morphological and physiological maturation by the onset of hearing [110, 112]. However, the intracellular signals regulating this pre-hearing functional maturation are still largely unknown. Regarding OHCs, it remains unclear whether they also exhibit spontaneous Ca^2+^ signals *in vivo*, and, if so, whether this activity is required for their maturation and the refinement of the type II fibres, analogous to the role proposed for IHCs. Recent experimental developments using live mice [9, 10, 124, 125] have the potential to address many of these remaining questions with unprecedented resolution. This approach will be instrumental in bridging the current understanding of single-cell physiology with systems-level data from the cochlea.
